# LncRNA BCYRN1 inhibits glioma tumorigenesis by competitively binding with miR-619-5p to regulate CUEDC2 expression and the PTEN/AKT/p21 pathway

**DOI:** 10.1038/s41388-020-01466-x

**Published:** 2020-09-25

**Authors:** Maolin Mu, Wanxiang Niu, Xiaoming Zhang, Shanshan Hu, Chaoshi Niu

**Affiliations:** 1 Department of Neurosurgery, The First Affiliated Hospital of USTC, Division of Life Sciences and Medicine, University of Science and Technology of China, Hefei, 230001 Anhui P.R. China; 2 Anhui Key Laboratory of Brain Function and Diseases, Hefei, 230001 Anhui P.R. China; 3 Anhui Provincial Stereotactic Neurosurgical Institute, Hefei, 230001 Anhui P.R. China

**Keywords:** CNS cancer, Prognostic markers

## Abstract

Glioma is the most common malignant tumor in the central nervous system. Altered long noncoding RNAs (lncRNAs) are playing regulatory roles in physiological and pathogenic processes in cancer. Here, we uncovered a differentially expressed lncRNA called brain cytoplasmic RNA 1 (BCYRN1), and elucidated its function and molecular mechanism in the progression and development of glioma. Three fresh tumor tissues from glioma patients and three normal brain tissues from craniocerebral trauma patients were prepared for high-throughput RNA sequencing. Differential RNA transcripts and BCYRN1 were identified by RT-qPCR in glioma samples and controls. CCK-8, colony formation assays, flow cytometry, TUNEL assays, cell migration assays, wound-healing assays, and xenograft model were established to investigate the biological function of BCYRN1 both in vitro and in vivo. Various bioinformatics analysis, dual-luciferase reporter assays, biotinylated RNA pulldown assays, and rescue experiments were conducted to reveal the underlying mechanisms of competitive endogenous RNAs (ceRNAs). 183 lncRNAs were identified with significant dysregulation in glioma and randomly selected differential RNAs were further confirmed by RT-qPCR. Among them, BCYRN1 was the most downregulated lncRNA, and its low expression positively correlated with glioma progression. Functionally, BCYRN1 overexpression inhibited cell proliferation, migration in glioma cell lines, whereas BCYRN1 depletion resulted in the opposite way. MiR-619-5p was further confirmed as the direct target of BCYRN1. Mechanistically, miR-619-5p specifically targeted the CUE domain containing protein 2 (CUEDC2), and BCYRN1/miR-619-5p suppressed glioma tumorigenesis by inactivating PTEN/AKT/p21 pathway in a CUEDC2-dependent manner. Overall, our data presented that the reduced expression of BCYRN1 was associated with poor patient outcome in glioma. BCYRN1 functioned as a ceRNA to inhibit glioma progression by sponging miR-619-5p to regulate CUEDC2 expression and PTEN/AKT/p21 pathway. Our results indicated that BCYRN1 exerted tumor suppressor potential and might be a candidate in the diagnosis and treatment of glioma.

## Introduction

Glioma is the most prevalent and malignant primary intracranial tumor. Glioblastoma multiforme (GBM) is one of the most lethal forms of human cancers with average survival rate of 12–15 months [1, 2]. With the aggravation of population aging, glioma is becoming a more and more serious threat to human health. Therefore, early diagnosis and treatment is a crucial medical problem. Due to the invasiveness nature of glioma, it is very difficult to be completely removed in the surgery [3]. What’s worse is this tumor resistant to radiation therapy and chemotherapy and easy to recurrence [1, 2]. Thus, molecular diagnosis, especially various biomarkers are necessary to be investigated during glioma occurrence and progression.

Dysregulated transcripts including mRNAs, microRNAs (miRNAs), lncRNAs, and circular RNAs (circRNAs) can be a primary feature in human cancers [4–13]. With the rapid development of high-throughput RNA sequencing and the wide application of bioinformatics, lncRNAs, as one subset of ncRNAs with the length >200 nucleotides, were identified to participate in diverse biological processes in cancer [7–9]. There are multiple kinds of mechanisms in cell development and disease for lncRNAs. For instance, Evx1as, a nuclear antisense lncRNA, was reported to regulate gene transcription *in cis* [14]. Xist is one of the most famous lncRNAs, which recruits blocking factors leading to X inactivation [15]. Metastasis associated lung adenocarcinoma transcript 1 (Malat1) is thought to form molecular scaffolds for ribonucleoprotein complexes, which was also reported to serve as a transcriptional regulator for numerous genes, including some genes involved in cancer metastasis, cell migration, and cell cycle [16, 17]. 5S-OT is a lncRNA overlapped with 5 S rRNA, which interacts with U2AF65 to modulate alternative splicing through RNA: RNA pairing [10]. Some lncRNAs act as the miRNA precursors to give rise to miRNAs to target genes [18]. Growing evidences have implicated that a number of lncRNAs functioned as ceRNAs to affect the occurrence and development of cancers. Taken as an example, H19, a well-known lncRNA, was highly regulated in glioma. H19 induced endothelial cell proliferation, migration, and tube formation in vitro, which were proved to exert an H19-mir-29a-VASH2 axis to regulate the tumorigenesis [19]. In glioma, a number of lncRNAs have been found to be significantly dysregulated, such as HOTAIR, TUG1, and ECONEXIN [20–24]. Nevertheless, the various functions and multiple mechanisms of lncRNAs in glioma have not been explored comprehensively.

MiRNAs are endogenously expressed small noncoding RNAs with a length of ~20–22 nucleotides, which play central roles in the ceRNA hypothesis [25]. MiRNAs are highly conserved across species, and regulate gene expression by binding to the 3′-untranslated region (3′-UTR) of target mRNAs. More and more miRNAs were demonstrated to play important roles in glioma [26–28].

In this study, we investigated the expression profiling of lncRNAs and identified a most downregulated lncRNA called BCYRN1 in glioma from RNA-seq. Lower BCYRN1 expression was related to poor prognosis of glioma patients. Furthermore, we found that BCYRN1 affected the proliferation, apoptosis, migration, invasion abilities in vitro and inhibited the tumor formation in vivo. In addition, our study revealed a mechanism that BCYRN1 competitively bind miR-619-5p to regulate glioma progression through inactivating the CUEDC2/PTEN/AKT/p21 pathway. Our results indicate that BCYRN1 exerts as a tumor suppressor in glioma and has the potential to be the promising therapeutic target for glioma diagnosis, therapy, and prognosis.

## Results

### Transcriptome profiling in glioma and characterization of lncRNA BCYRN1

**Fig. 1:**
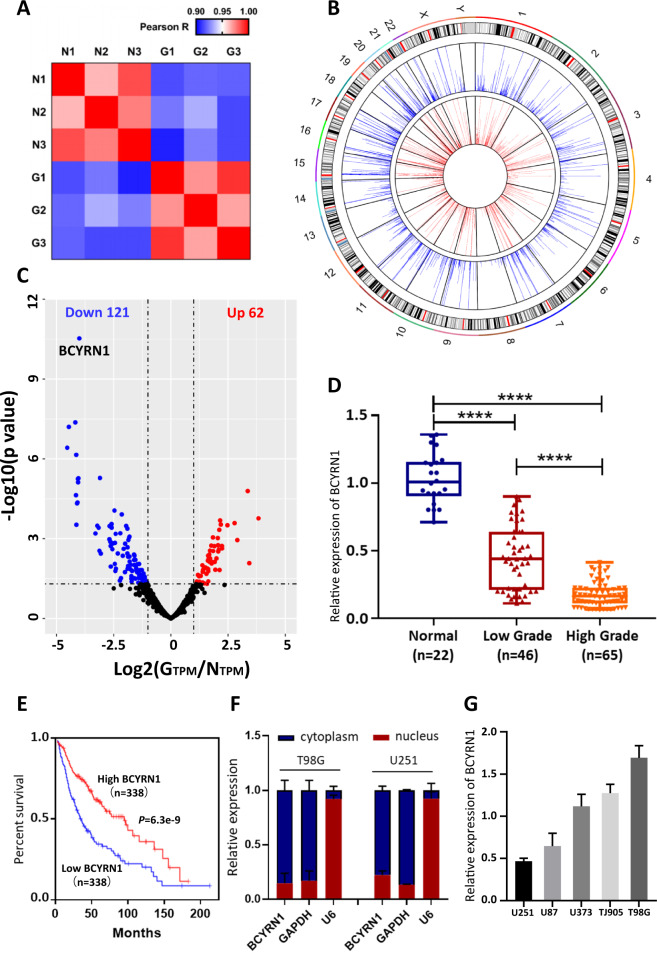
RNA-seq analysis of glioma and characterization of BCYRN1. **a** Heatmap of correlation for all transcripts between each sample. N normal tissue, G glioma tissue. **b** Circos plots of lncRNAs in the human genome (hg19). The outer tracks represent the cytoband ideogram of chromosome. For the two tracks, the outer one (blue) represents the levels of lncRNAs in normal tissues and the inner one (red) represents the levels in glioma tissues. **c** Volcano plots performed the expressed lncRNAs. Blue and red dots represent downregulated and upregulated lncRNAs respectively in glioma tissues compared with normal tissues. Black dots indicate the lncRNAs with no significant differences. **d** Expression level of BCYRN1 was identified in 22 normal tissues and 46 low grade glioma tissues and 65 high grade glioma tissues by RT-qPCR. **e** Kaplan–Meier plots of overall survivals in glioma patients with high (*n* = 338) and low (*n* = 338) levels of BCYRN1. **f** Levels of BCYRN1 in the nuclear and cytoplasmic fractions of T98G and U251 cells. **g** RT-qPCR analysis of the relative expression of BCYRN1 in five glioma cell lines. Data were from three independent experiments. In (**d**), error bars, S.E.M. ****p < 0.0001 by two-tailed Student’s *t* test. In (**e**), error bars, S.E.M. *P* value was calculated by Mantel–Cox log rank test.

In order to obtain a comprehensive and profound view of lncRNA transcripts in glioma, we performed high-throughput RNA sequencing using three tissue samples from glioma patients (diagnosed by enhanced MRI and pathology) and three normal brain tissues from craniocerebral trauma patients (Fig. s1a, b). To ensure the authenticity and validity of RNA-seq data, we performed the Pearson’s correlation coefficient analysis of all transcripts between each sample (Fig. 1a). Tumor tissues or control tissues themselves demonstrated the higher correlation compared to each other (Fig. 1a). Notably, we found the expression levels of some previously reported mRNAs and lncRNAs were significantly dysregulated in our data (Table S1). Circos plots globally and genome-widely displayed 1344 kinds of detected lncRNAs in glioma tissues and controls (Fig. 1b). Volcano plots were performed for all expressed lncRNAs (Fig. 1c). There were 183 lncRNAs showed significantly dysregulated in glioma, in which 121 were downregulated while 62 were upregulated (Fig. 1c, Fig. s1c). Hierarchical cluster analysis was also constructed to reveal the differential expression of lncRNAs in glioma tissues and controls (Fig. s1c). To validate the results, 6 lncRNAs were randomly selected for RT-qPCR analysis. THBS3, ATP6VOE, and LINC01235 were significantly upregulated while USP32P2, RTN3, and LINC00294 were markedly downregulated in glioma compared to controls (Fig. s1d). All these validations were consistent with the RNA-seq data. BCYRN1, as the most downregulated lncRNA in glioma, was chosen for further studies.

Next, we confirmed the downregulated expression of BCYRN1 in 22 normal tissues and 111 glioma tissues (46 Grade I and II, 65 Grade III and IV) by RT-qPCR, and found that the expression of BCYRN1 was significantly lower in glioma tissue, especially reduced with advanced glioma grade (Fig. 1d, Table 1). In addition, we assessed the correlation between BCYRN1 expression and prognosis of glioma patients. Kaplan–Meier analysis of TCGA database demonstrated that patients with lower BCYRN1 expression were more likely to be poor overall survivals (Fig. 1e). Taken together, these data suggested that BCYRN1 downregulation was common in glioma tissues and was correlated with poor prognosis.

**Table 1:** The relationship of lncBCYRN1 and clinical characteristics in 111 glioma patients.

Variable	LncBCYRN1		*p* value
	High (*n* = 48)	Low (*n* = 63)	
Sex			0.685
Male	27	33	
Female	21	30	
Age(year)			0.120
≤45	23	21	
>45	25	42	
WHO grade			0.012
I	16	8	
II	13	12	
III	11	19	
IV	8	24	
Location			0.709
Frontal	12	17	
Parietal	10	13	
Occipital	9	7	
Temporal	17	26	
Recurrence			0.881
NO	46	60	
Yes	2	3	
Histology			0.022
Pilocytic Astrocytoma	5	2	
Oligodendroglioma	16	8	
Oligoastrocytic	6	13	
Astrocytome	5	8	
Anaplastic Astrocytoma	10	12	
Glioblastoma	6	20	

### BCYRN1 inhibits glioma progression in vitro and in vivo

To identify cellular localization of BCYRN1, RT-qPCR analysis of nuclear and cytoplasmic RNAs was carried out to show that BCYRN1 was preferentially located in the cytoplasm (Fig. 1f), consistent with the subcellular fractionation in other cancer types [29–31]. Furthermore, five kinds of glioma cell lines were tested the endogenous expression level of BCYRN1. Based on the results, T98G and U251 were selected for the loss-of-function and gain-of-function assays in vitro (Fig. 1g).

**Fig. 2:**
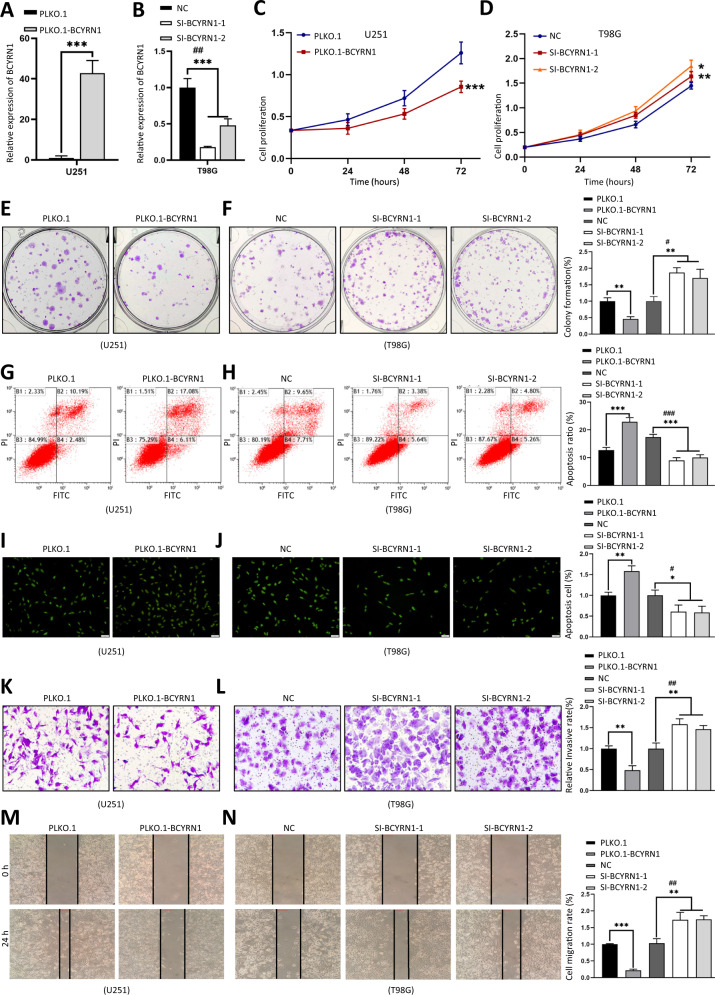
BCYRN1 inhibits glioma progression in vitro. **a** Overexpression level of BCYRN1 in U251 cells. **b** Expression level of BCYRN1 in T98G cells treated with two independent siRNAs. **c**, **d** CCK-8 analysis of glioma cells with overexpression or silencing of BCYRN1. **e**, **f** Colony formation assays of glioma cells with overexpression or silencing of BCYRN1. **g**, **h** Flow cytometry of glioma cells with overexpression or silencing of BCYRN1. **i**, **j** TUNEL assays of glioma cells with overexpression or silencing of BCYRN1. Scale bars, 50 μm. **k**, **l** Transwell assays of glioma cells with overexpression or silencing of BCYRN1. **m**, **n** Wound-healing assays of glioma cells with overexpression or silencing of BCYRN1. Error bars, S.E.M. from three independent experiments. *P < 0.05; **P < 0.01; ***P < 0.001 by two-tailed Student’s *t* test. #P was used to show the significance between NC and Si-BCYRN1-2.

To explore the functional roles of BCYRN1, we overexpressed the full length in U251 and T98G cells (Fig. 2a, Fig. s2a). Two short interfering RNAs against different region of BCYRN1 were performed, which effectively knockdown BCYRN1 expression level in T98G and U251 cells (Fig. 2b, Fig. s2b). CCK-8 and colony formation assay revealed that overexpression of BCYRN1 inhibited cell growth and colony formation (Fig. 2c, e, Fig. s2c, e). On the other hand, flow cytometry and TUNEL assay demonstrated overexpression of BCYRN1 induced cell apoptosis (Fig. 2g, i, Fig. s2g, i). In addition, transwell and wound-healing assay displayed that upregulation of BCYRN1 significantly reduced cell migratory capacity (Fig. 2k, m, Fig. s2k, m). In contrast to overexpression, ablation of BCYRN1 promoted cell growth, colony formation, migration, and repressed cell apoptosis in U251 and T98G cells (Fig. 2d, f, h, j, l, n, Fig. s2d, f, h, j, l, n).

**Fig. 3:**
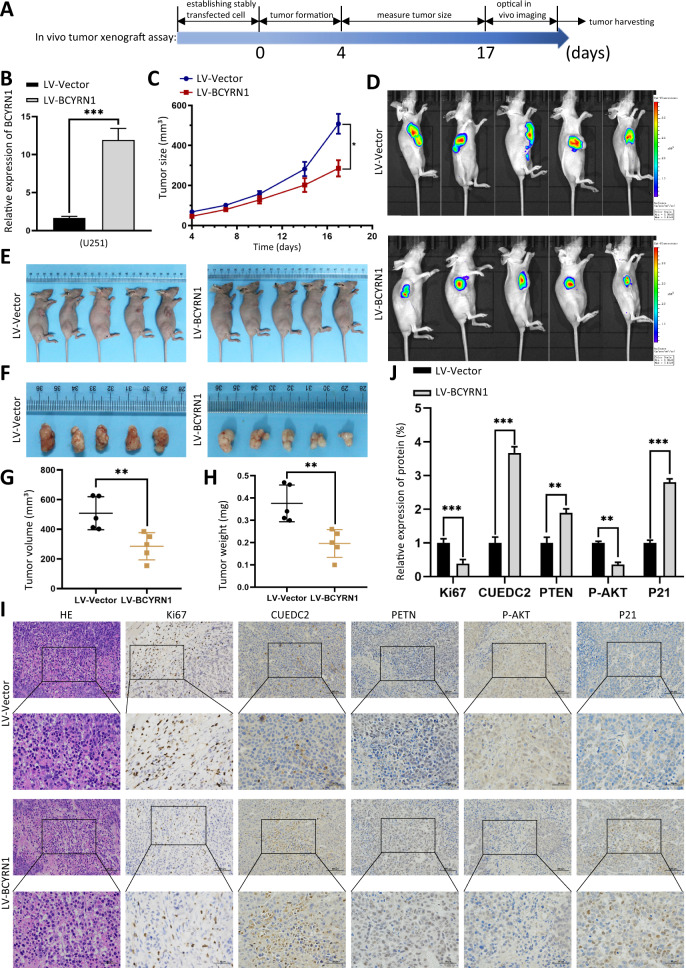
BCYRN1 inhibits glioma tumor formation in vivo. **a** Illustration of the tumor transplantation timelines when treated with BCYRN1 overexpression lentivirus. The divergent arrows indicate the different stages. **b** Expression level of BCYRN1 in U251 cells after transduction with overexpression lentivirus. **c** The tumor sizes were measured. **d** Bioluminescence imaging of xenograft tumors in mice was shown. **e** Stable U251 cells (1 × 10^7^ per mouse, *n* = 5 each group) were subcutaneously injected into BALB/c nude mice (two groups) to establish subcutaneous xenograft tumors. **f** The dissected tumors were photographed. **g**, **h** The tumor volumes and weights were measured. **i**, **j** IHC staining revealed the expression of Ki67, CUEDC2, PTEN, P-AKT, P21 when treated with BCYRN1 overexpression lentivirus. Scale bars, 50 μm. Error bars, S.E.M. from three independent experiments. *P < 0.05; **P < 0.01; ***P < 0.001 by two-tailed Student’s *t* test.

In order to investigate regulatory roles of BCYRN1 on tumor growth in vivo, we established U251 stable cell line with lentivirus-BCYRN1 to overexpress BCYRN1. The procedure of in vivo tumor xenograft assay was showed (Fig. 3a). Overexpression efficiency of BCYRN1 was revealed by RT-qPCR assays (Fig. 3b). We observed that tumor size and weights were reduced in the LV-BCYRN1 group compared with those in the LV-vector group (Fig. 3c–h). IHC staining revealed that Ki-67 expression was downregulation upon BCYRN1 overexpression in dissected tumors (Fig. 3i, j). Collectively, these results in vitro and in vivo strongly suggested that BCYRN1 was a potential tumor suppressor in glioma.

### BCYRN1 acts as a ceRNA for miR-619-5p in glioma

**Fig. 4:**
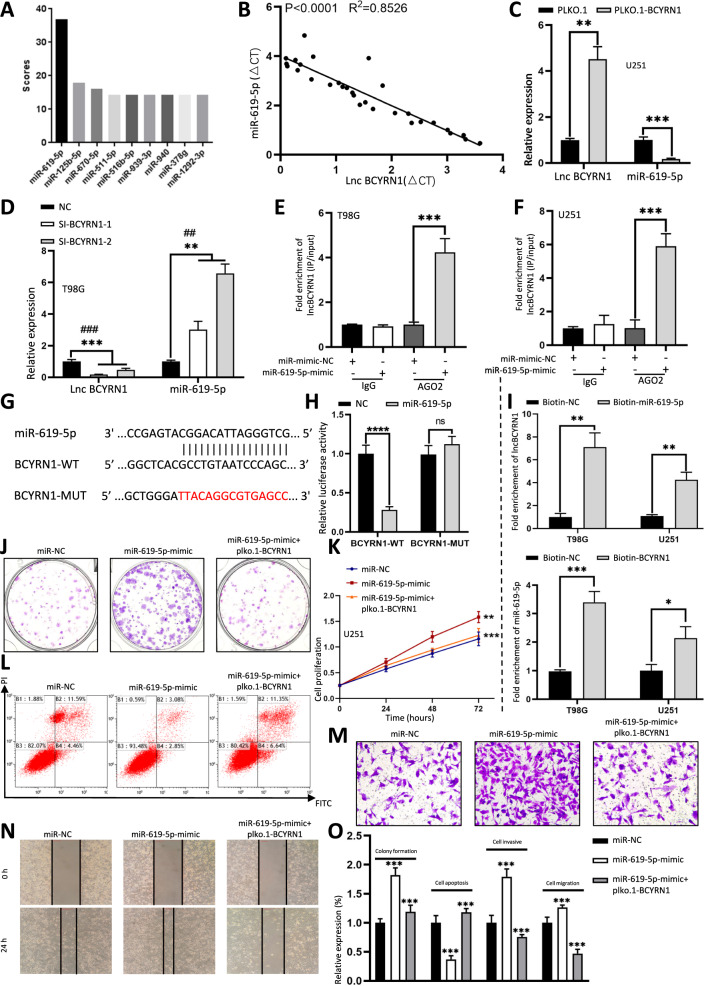
BCYRN1 inhibits glioma progression by directly binding to miR-619-5p. **a** Blast scores of miRNAs aligned to BCYRN1. **b** The Pearson correlation between lncRNA BCYRN1 level and miR-619-5p level was measured in the same set of glioma tissues (2 Grade I, 9 Grade II, 5 Grade III, 14 Grade IV). The ΔCt values (normalized to GAPDH) were subjected to Pearson correlation analysis (*R*^2^ = 0.8526, *P* < 0.0001). **c** Expression level of miR-619-5p in U251 cells after transfection with overexpression plasmids of BCYRN1. **d** Expression level of miR-619-5p in T98G cells treated with siRNAs of BCYRN1. **e**, **f** RIP was performed using AGO2 antibody in T98G and U251 cell lines transfected with miR-619-5p mimics or mimics NC, then the enrichment of BCYRN1 was detected. **g** The binding sites of BCYRN1 and miR-619-5p. **h** Luciferase reporter activity of wild-type (WT) or mutated (MUT) BCYRN1 in 293 T cells co-transfected with miR-619-5p mimics. **i** MiR-619-5p was pulled down with biotinylated BCYRN1 in T98G and U251 cells; BCYRN1 was pulled down with biotinylated miR-619-5p in T98G and U251 cells. **j** Colony formation assays in U251 cells transfected with miR-619-5p mimic alone or co-transfection with BCYRN1. **k** CCK-8 analysis in U251 cells transfected with miR-619-5p mimic alone or co-transfection with BCYRN1. **l** Flow cytometry in U251 cells transfected with miR-619-5p mimic alone or co-transfection with BCYRN1. **m** Transwell assays in U251 cells transfected with miR-619-5p mimic alone or co-transfection with BCYRN1. **n** Wound-healing assays in U251 cells transfected with miR-619-5p mimic alone or co-transfection with BCYRN1. **o** Relative expression of (**j**), (**l**), (**m**), (**n**). Error bars, S.E.M. from three independent experiments. *P < 0.05; **P < 0.01; ***P < 0.001; ****p < 0.0001 by two-tailed Student’s *t* test. #P was used to show the significance between NC and Si-BCYRN1-2.

As shown in Fig. 1f, BCYRN1 was preferentially located in the cytoplasm. It has been shown that cytoplasmic lncRNAs can act as miRNAs sponge to regulate downstream targets [32–34]. In order to explore whether BCYRN1 might also function as the ceRNA mechanism, we searched candidate miRNAs by miRanda prediction. We observed that multiple miRNAs were able to align to lncRNA BCYRN1 and blast score of miR-619-5p was the highest (Fig. 4a). MiR-619-5p showed a severe overexpression in 30 glioma samples compare to control samples (Fig. s3a, Table S2). MiR-619-5p expression was the highest in U251 and was the lowest in T98G, which was inverse to BCYRN1 in the two cell lines (Fig. s3b). Furthermore, the miR-619-5p level was negatively correlated with the lncRNA BCYRN1 level in the glioma tissues (Fig. 4b). Overexpression of BCYRN1 led to the significant downregulation of miR-619-5p, whereas its silencing resulted in the upregulation of miR-619-5p (Fig. 4c, d). AGO2 immunoprecipitation (IP) was performed to determine whether BCYRN1 served as a component for AGO2 and miR-619-5p complex. It turned out BCYRN1 was enriched in AGO2 IP of miR-619-5p transfected cells (Fig. 4e, f). To verify that BCYRN1 could bind to miR-619-5p, luciferase reporters containing wild-type (BCYRN1-WT) and mutated miR-619-5p binding sites (BCYRN1-MUT) were constructed. We observed that luciferase activity of BCYRN1-WT was significantly reduced after co-transfection of miR-619-5p mimic, but luciferase activity of BCYRN1-MUT did not change, which suggested that miR-619-5p was a target of BCYRN1 in a sequence-specific manner (Fig. 4g, h). In addition, biotin-coupled BCYRN1 successfully pulled down the competitive binding of miR-619-5p, while biotin-labeled miR-619-5p also pulled down the binding of BCYRN1 (Fig. 4i, Fig. s3c). All these findings suggested BCYRN1 acted as a ceRNA for miR-619-5p in glioma.

We further tested the fundamental role of miR-619-5p itself in glioma. MiR-619-5p expression was increased or decreased after T98G and U251 cells were transfected with miR-619-5p mimic or inhibitor, respectively (Fig. s3d, e). Overexpression of miR-619-5p induced cell growth and migration, inhibited cell apoptosis (Fig. s3f, h, j). On the other hand, ablation of miR-619-5p suppressed cell growth and migration, promoted cell apoptosis (Fig. s3g, i, k). More importantly, upregulation of BCYRN1 could partially rescue the promotive effects of miR-619-5p on cell growth and migration, and the reductive effect of miR-619-5p on cell apoptosis in glioma (Fig. 4j–o). Meanwhile, ablation of BCYRN1 could partially rescue the reductive effects of miR-619-5p on cell growth and migration, and the inductive effect of miR-619-5p on cell apoptosis in glioma (Fig. s4). Taken together, our results indicated BCYRN1 served as a sponge for miR-619-5p in the regulation of glioma progression.

### BCYRN1 inhibits glioma progression by targeting miR-619-5p/CUEDC2

**Fig. 5:**
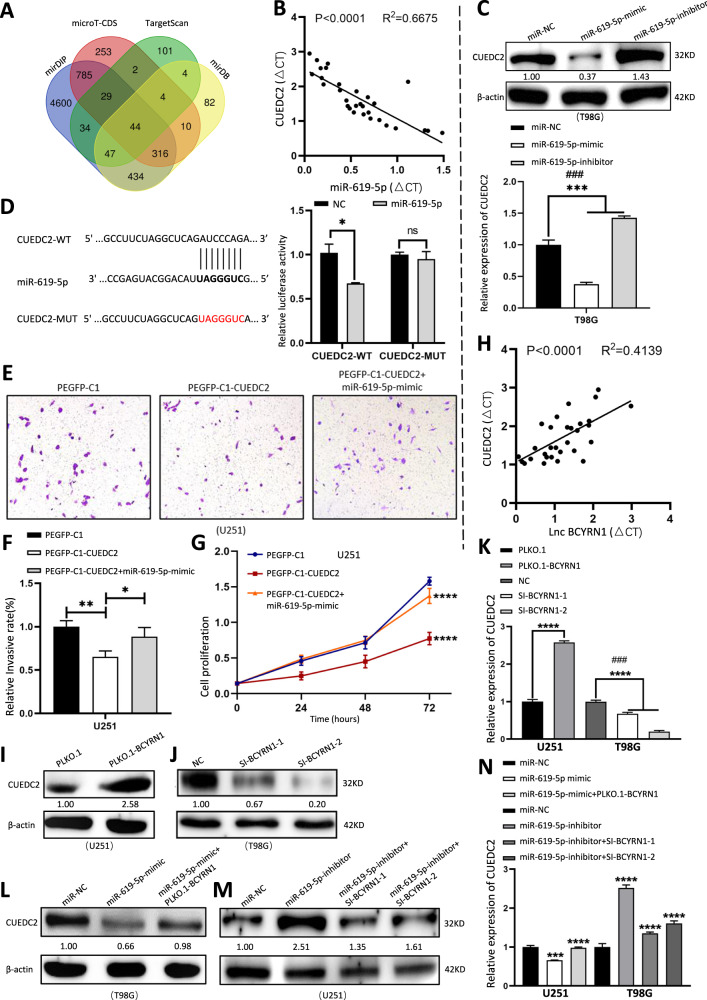
CUEDC2 is a direct target of miR-619-5p. **a** Identification of 44 commonly altered targeted mRNAs of miR-619-5p from four public profile datasets (mirDIP, microT-CDS, TargetScan, mirDB). **b** The Pearson correlation between CUEDC2 level and miR-619-5p level was measured in the same set of glioma tissues (2 Grade I, 9 Grade II, 5 Grade III, 14 Grade IV). The ΔCt values (normalized to GAPDH) were subjected to Pearson correlation analysis (*R*^2^ = 0.6675, *P* < 0.0001). **c** The mRNA and protein expression level of CUEDC2 in T98G cells treated with miR-619-5p mimic or inhibitor. **d** Luciferase reporter activity of wild-type (WT) or mutated (MUT) CUEDC2 in 293 T cells co-transfected with miR-619-5p mimics. **e**, **f** Transwell assays in U251 cells transfected with CUEDC2 alone or co-transfection with miR-619-5p mimics. **g** CCK-8 analysis in U251 cells transfected with CUEDC2 alone or co-transfection with miR-619-5p mimics. **h** The Pearson correlation between lncRNA BCYRN1 level and CUEDC2 level was measured in the same set of glioma tissues (2 Grade I, 9 Grade II, 5 Grade III, 14 Grade IV). The ΔCt values (normalized to GAPDH) were subjected to Pearson correlation analysis (*R*^2^ = 0.4139, *P* < 0.0001). **i**, **k** The mRNA and prote**i**n expression level of CUEDC2 in U251 cells treated with transfection of BCYRN1 overexpression plasmid. **j**, **k** The mRNA and protein expression level of CUEDC2 in T98G cells treated with BCYRN1 siRNAs. **l**, **n** The mRNA and protein expression level of CUEDC2 in T98G cells treated with miR-619-5p mimic alone or co-transfection with BCYRN1. **m**, **n** The mRNA and protein expression level of CUEDC2 in U251 cells treated with miR-619-5p inhibitor alone or co-transfection with BCYRN1 siRNAs. Error bars, S.E.M. from three independent experiments. *P < 0.05; **P < 0.01; ***P < 0.001; ****p < 0.0001 by two-tailed Student’s *t* test. #P was used to show the significance between NC and Si-BCYRN1-2 or miR-NC and miR-619-5p-inhibitor.

MiRNAs exert various biological functions by targeting mRNAs. To explore the mechanism of BCYRN1/miR-619-5p in glioma progression, we performed comprehensive bioinformatic analysis using four datasets and found that 44 mRNAs might be the potential targets of miR-619-5p including CUEDC2 (Fig. 5a), which was reported to be a tumor suppressor in glioma [35]. CUEDC2 showed an obvious downregulation in 30 glioma samples compared to control samples (Fig. s5a, Table S2). Kaplan–Meier analysis of TCGA database demonstrated that patients with lower CUEDC2 expression were more likely to be poor overall survivals (Fig. s5b).

Importantly, the miR-619-5p level was inversely correlated with the CUEDC2 level in the glioma tissues (Fig. 5b). Furthermore, both the mRNA and protein levels of CUEDC2 were decreased or increased after treated with miR-619-5p mimic or inhibitor, respectively (Fig. 5c). Then we used TargetScan database to predict the binding site of miR-619-5p on CUEDC2 3′-UTR region (Fig. 5d). Luciferase assays were performed to verify a direct interaction between miR-619-5p and CUEDC2. CUEDC2 3′-UTR sequence containing the seed region of miR-619-5p (CUEDC2-WT) and mutated miR-619-5p binding sites (CUEDC2-MUT) were constructed and transfected into 293 T cell line. The results showed that luciferase activity of CUEDC2-WT was significantly reduced after co-transfection of miR-619-5p mimic, but luciferase activity of CUEDC2-MUT did not change (Fig. 5d). Upregulation of CUEDC2 could partially rescue the promotive effects of miR-619-5p on cell growth and migration (Fig. 5e–g), while inhibition of CUEDC2 could partially rescue the reductive effects of miR-619-5p on cell growth and migration in glioma (Fig. s5c-e). These findings indicated that CUEDC2 was the target of miR-619-5p.

We found that the CUEDC2 level was positively correlated with the BCYRN1 level in the glioma tissues (Fig. 5h). To verify whether the CUEDC2 gene was modulated by BCYRN1, overexpression and knockdown of BCYRN1 were performed. The results showed that both the mRNA and protein levels of CUEDC2 were decreased or increased upon BCYRN1 overexpression or knockdown, respectively (Fig. 5i–k). Upregulation of BCYRN1 could rescue the inhibitory effect of miR-619-5p mimic on CUEDC2 expression, while knockdown of BCYRN1 could partially rescue the induction of miR-619-5p inhibitor on CUEDC2 expression both in mRNA and protein level (Fig. 5l–n). Taken together, these data provided evidences that inhibition glioma progression of BCYRN1 was primarily dependent on the miR-619-5p/ CUEDC2 axis.

### BCYRN1/miR-619-5p/CUEDC2 axis negatively regulates glioma via PTEN/AKT/p21 pathway

**Fig. 6:**
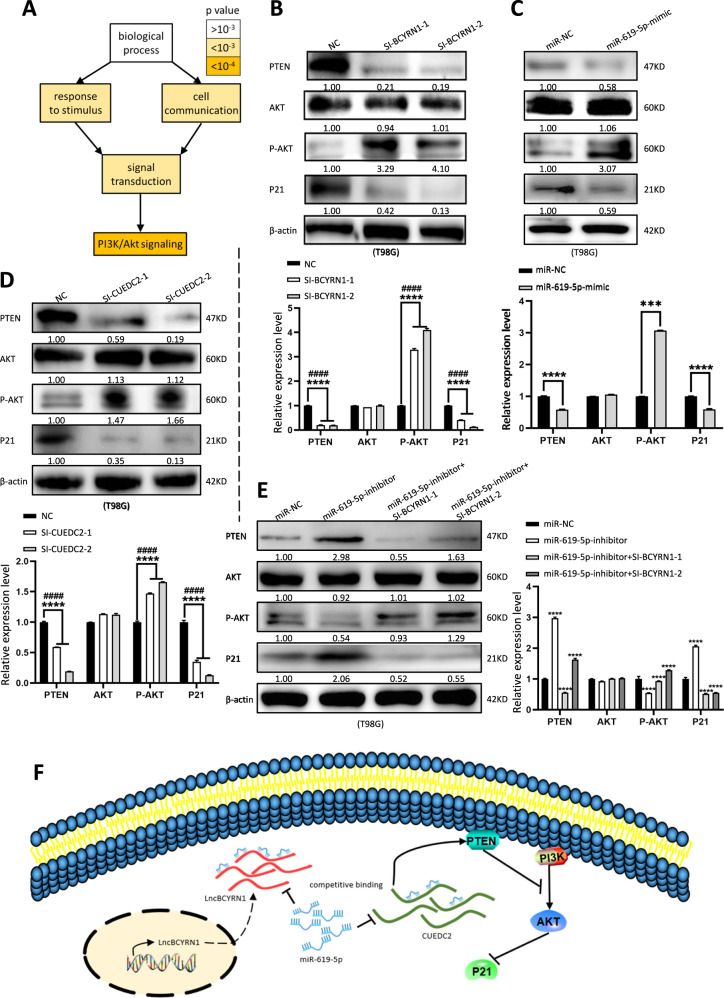
BCYRN1 inhibits glioma progression through CUEDC2/PTEN/Akt/P21 signaling pathway. **a** GO analysis and annotation of 44 targeted mRNAs in biological processes. **b** The protein expression levels of PI3K/Akt signaling related proteins in T98G cells treated with BCYRN1 siRNAs. **c** The protein expression levels of PI3K/Akt signaling related proteins in T98G cells treated with miR-619-5p mimic. **d** The protein expression levels of PI3K/Akt signaling related proteins in T98G cells treated with CUEDC2 siRNAs. **e** The protein expression level of PI3K/Akt signaling related proteins in T98G cells treated with miR-619-5p inhibitor alone or co-transfection with BCYRN1 siRNAs. **f** Hypothesis diagram illustrates function and mechanism of BCYRN1 in glioma. Error bars, S.E.M. from three independent experiments. ****p < 0.0001 by two-tailed Student’s *t* test. #P was used to show the significance between NC and Si-BCYRN1-2 or NC and Si-CUEDC2-2.

Gene Ontology (GO) biological process enrichment analyses of the 44 mRNAs in the network demonstrated the functional associations with several important pathways, including biological processes such as response to stimulus, cell communication, signal transduction, especially PI3K/Akt signaling pathway (Fig. 6a). It is well established that PI3K/Akt signaling played important regulatory role in glioma, and we examined the pathway related proteins by western blot. Knockdown BCYRN1 showed activation of AKT signaling, increased expression of Phosphorylated-AKT (P-AKT) protein, decreased expression of PTEN and P21 (Fig. 6b). In addition, overexpression of BCYRN1 in vivo led to decrease of P-AKT and increase of CUEDC2, PTEN, and P21(Fig. 3i, j). As expected, overexpression of miR-619-5p, as well as knockdown CUEDC2, also activated AKT signaling, increased expression of P-AKT protein, decreased expression of PTEN and P21(Fig. 6c, d). Therefore, downregulation of BCYRN1 could rescue the promotive effect of miR-619-5p inhibitor on PI3K/Akt signaling protein expression level (Fig. 6e). In addition, CUEDC2 induced abnormal expression level of PI3K/Akt signaling related proteins reversed with miR-619-5p (Fig. s5f-m). In conclusion, our results indicated that PTEN/AKT signaling participated in BCYRN1-mediated inhibition of glioma progression via miR-619-5p/CUEDC2 axis.

## Discussion

Transcriptome profiling is an effective approach for the global view of cancers [36]. More importantly, it is crucial to identify tumor suppressors and oncogenic lncRNAs then elucidate their functions and mechanisms. In this study, we identified hundreds of markedly altered transcripts including 183 lncRNAs through RNA-seq in glioma. Interestingly, altered transcripts were downregulated in glioma compared to controls in our present study as well as other reports [37]. Among them, BCYRN1 was the most significantly downregulated lncRNA in glioma. Gain-of-function and loss-of-function experiments demonstrated that BCYRN1 inhibited the occurrence and development of glioma in vitro and in vivo. BCYRN1 exhibited its function as a ceRNA that competitively bind to miR-619-5p, then regulated target gene CUEDC2 expression and the PTEN/AKT/p21 pathway (Fig. 6f).

LncRNAs are emerging as important regulators in different disease processes including cancer [34, 38–42]. Recently, growing numbers of lncRNAs have been found to be dysregulated in glioma. HOX transcript antisense RNA (HOTAIR) was overexpressed in GBM, crucial to sustain tumor cell proliferation, and regulated by BET bromodomain protein [43]. ECONEXIN is a potential oncogene that regulates TOP2A by sponging miR-411-5p in glioma [23]. In this study, we found that lncRNA BCYRN1 was significantly lower in glioma than normal brain tissues and it was negatively correlated with glioma grade. BCYRN1, termed as BC200, has been found in a variety of cancers as previous studies. Either as an oncogenic lncRNA or a tumor suppressor, BCYRN1 was reported to exert significant biological function in cancers such as NSCLC, ovarian cancer, and gastric cancer [31, 44–48]. However, as a lncRNA derived from brain cytoplasm, the expression and function of BCYRN1 in glioma remain unknown. We believe that it is the first comprehensive characterization of BCYRN1 function and mechanism in glioma. Our results revealed the clinical significance of BCYRN1 and indicated that BCYRN1 might be a candidate in diagnosis and treatment of glioma.

It has been extensively reported that cytoplasmic lncRNAs could function as miRNA sponge to modulate mRNA stability or translation and affect related signaling pathways [49–53]. Bioinformatic analyses and luciferase reporter assays verified that miR-619-5p was the binding target of BCYRN1. MiR-619-5p has been reported to play important roles in several diseases [54, 55]. For example, miR-619-5p was markedly downregulated in colorectal carcinoma and predicted to be a prognostic indicator of CRC patients [55]. However, the regulatory roles of miR-619-5p in glioma remain unclear. In this study, we found that miR-619-5p could promote cell proliferation and migration in glioma and sponged by BCYRN1. Of course, the ceRNA network has its own limitation, and additional studies of lncRNAs as ceRNA in human cancers are needed.

BCYRN1 shared common miR-619-5p binding sites with CUEDC2. CUEDC2 played critical roles in many biological processes, such as cell cycle, inflammation, and tumorigenesis [56–62]. As a multifunctional protein, the function of CUEDC2 in cancers is debated. Li et al. demonstrated that CUEDC2 acted as a tumor suppressor and inhibited the tumorigenicity of glioma by inactivating STAT3 and NF-κB signaling pathways [35]. While Wang et al. found that CUEDC2 contributed to cisplatin-based chemotherapy resistance by regulating p38 MAPK signaling and was a promising biomarker and therapeutic target of cisplatin resistance in ovarian serous carcinoma [62]. In this study, we showed that CUEDC2 was downregulated in glioma and the lower expression was more likely to be poor overall survivals, which supported that reduced level of BCYRN1 was correlated with poor prognosis.

Finally, we explored downstream targets of CUEDC2 essential for BCYRN1-mediated tumor suppressor function. Knockdown BCYRN1, as well as overexpression of miR-619-5p, activated AKT signaling, increased expression of P-AKT protein, decreased expression of PTEN and P21 through downregulating CUEDC2. In fact, PTEN/AKT signaling has been widely reported to play vital regulatory roles in cancers including glioma [63–65]. Our findings demonstrated that the role of the regulatory network between BCYRN1/miR-619-5p/CUEDC2 in glioma was achieved by affecting PTEN/AKT signaling pathway activity. Based on the fact that inactivation of the PTEN signaling pathway, frequently found to be disrupted in human glioma [66], we suspected that there were some other miRNAs or other mRNA targets of BCYRN1, contributing to the effect of BCYRN1, when PTEN was lost. However, other mechanisms underlying the downregulation of BCYRN1 in glioma need to be further investigated in our future studies.

In conclusion, we performed high-throughput RNA sequencing to uncover a downregulated lncRNA BCYRN1 in glioma and verified with clinical glioma samples. BCYRN1 inhibited glioma progression in vitro and in vivo. Our study revealed a mechanism for BCYRN1 that competitively bind miR-619-5p, in turn, suppressed glioma proliferation, migration through inactivating the CUEDC2/PTEN/AKT/p21 pathway. Overall, our study provided a new perspective to identify the potential biomarkers and therapeutic targets for glioma.

## Materials and methods

### Clinical samples preparation

All fresh glioma patient tumor samples and normal tissues were collected from Department of Neurosurgery of The First Affiliated Hospital of University of Science and Technology of China, which was approved by the Human Research Ethics Committee of the hospital. In this study, we obtained the written informed consent from each patient. A total of 111 resected brain tumors were obtained from May 2015 to May 2019, and all tumor tissues were clinically and histopathological diagnosed as glioma (WHO I/II 46, WHO III/IV 65). Normal brain tissues obtained from 22 patients with brain tissue resection due to craniocerebral injury during the period from May 2015 to May 2019. All samples were rinsed with PBS after operation, and then were cut into small pieces with RNAhold (TransGen) immersed. All samples were stored in −80 °C for the following experiments.

### Cell culture

Glioma cell lines U251, T98G, U87, TJ905 and U373 in the experiments were purchased from the ATCC. All cell lines were cultured in DMEM medium (HyClone) containing 10% FBS (Clark Bioscience) and stored in a humidified incubator at 37 °C with 5% CO2.

### Plasmids construction and cell transfection

Our plasmids were constructed with restriction-enzyme digestion and ligation or with recombinant methods (Vazyme). Full length of BCYRN1 was inserted into Plko.1 vector and CDS of CUEDC2 was inserted into PEGFP-C1 vector, respectively. SiRNAs, miRNA mimic and inhibitors were purchased from RiboBio. Plasmid and siRNAs transfection were conducted with Lipofectamine 3000 (Invitrogen) according to the manufacturer’s protocol. The sequences are showed in Table S3.

## Supplementary information

Supplementary Information

Figure S1

Figure S2

Figure S3

Figure S4

Figure S5

Table S1

Table S2

Table S3
