# Correction: LncRNA BCYRN1 inhibits glioma tumorigenesis by competitively binding with miR-619-5p to regulate CUEDC2 expression and the PTEN/AKT/p21 pathway

**DOI:** 10.1038/s41388-021-01990-4

**Published:** 2021-08-16

**Authors:** Maolin Mu, Wanxiang Niu, Xiaoming Zhang, Shanshan Hu, Chaoshi Niu

**Affiliations:** 1 Department of Neurosurgery, The First Affiliated Hospital of USTC, Division of Life Sciences and Medicine, University of Science and Technology of China, Hefei, 230001 Anhui P.R. China; 2 Anhui Key Laboratory of Brain Function and Diseases, Hefei, 230001 Anhui P.R. China; 3 Anhui Provincial Stereotactic Neurosurgical Institute, Hefei, 230001 Anhui P.R. China

**Keywords:** CNS cancer, Prognostic markers

Correction to: *Oncogene* 10.1038/s41388-020-01466-x

Following publication of this article, it was noted that the incorrect image for PEGFP-C1-CUEDC2+miR-619-5p-mimic was presented in Fig. 5e. The correct figure, based on the original raw data, is shown below. This correction does not change the results or conclusions of this paper.

The original article has been corrected.

**Figure:**
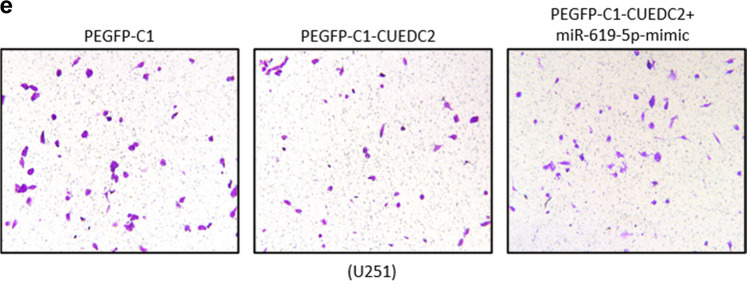
Fig. 5e.

