# Supplementary material for: LncRNA BCYRN1 inhibits glioma tumorigenesis by competitively binding with miR-619-5p to regulate CUEDC2 expression and the PTEN/AKT/p21 pathway
Source: Oncogene. 2020 Sep 25;39(45):6879–92. doi: 10.1038/s41388-020-01466-x (PMC7644463; doi:10.1038/s41388-020-01466-x)
Supplement: Supplementary file 1 — Supplementary Information [file 41388_2020_1466_MOESM1_ESM.docx]

**Supplementary Materials and Methods**

**Total RNA Isolation and Library Preparation**

Total RNA was extracted by using Trizol reagent (Invitrogen) according to the manufacturer’s instructions. The concentration and quality of total RNA were measured by Nanodrop and verified by gel electrophoresis. The Ribo-minus transcriptome libraries were constructed with TruSeq Ribo Profile Library Prep Kit (Illumina), according to the manufacturer’s instructions. The libraries were then subjected to 151-nt paired-end sequencing generating a depth of ~100 million read pairs with an Illumina Nextseq 500 system (Novogene, China).

**Reverse Transcription and Real-Time Quantitative PCR**

cDNA was prepared using GoScript Reverse Transcription System (Promega) according to the manufacturer’s protocol. Quantitative real-time PCR was performed with TransStart Green qPCR SuperMix (TransGen) on ABI7500 real-time PCR system (Applied Biosystems) according to standard procedures. All primers are shown in Table S3.

**Transcriptome Data Analysis**

The adapters were first trimmed with cutadapt to obtain clean reads and the left reads were then aligned to the human genome (hg19) with bowtie2 allowing one mismatch. The continuous or non-continuous mapped reads were subjected for the following mRNA and lncRNA analyses. The linear expression levels were evaluated with TopHat2 and Cufflinks followed by the annotation references of Refseq. The differentially expressed mRNA and lncRNA were determined by DEseq2 with the corresponding cutoff (P value <0.001, RPKM ≥10, |log2(fold change)| ≥1 for mRNA and P value <0.05, RPKM ≥1, |log2(fold change)| ≥1 for lncRNA).

**Cell proliferation assay**

For the cell proliferation ability, 24 h after transfection, cells were seeded into 96-well culture plates at a density of 4 ×10^3^ cells per well to incubate for 0, 24, 48, and 72 h. Cell Counting Kit-8 (CCK-8; biosharp) was added to each well and incubated for 4 h in a culture environment with 5% CO2 at 37 °C. The absorbance of each well was measured at 450 nm with the Infinite M200 (Tecan, Switzerland).

**Cell invasion and migration assays**

The cell migration and invasion abilities were determined using transwell chamber (8 μm, 24-well insert; Costar). In invasion assay, matrigel (BD Biosciences) was diluted with serum-free medium (1:40), mixed and used to coat the insert chamber membrane. Then, cells (1 × 10^4^) with 48 h transfection in serum-free medium were added to the upper chamber, and medium containing 10% FBS were added to the lower. For migration assay, it is not necessary to coat the insert chamber membrane with diluted matrigel. Cells were incubated for 48 h in a cell culture incubator with 4% formaldehydum polymerisatum. 0.1% crystal violet was used for the fixation and staining of the invading or migrating cells. Finally, the number of cells that migrated or invaded into the lower chambers was counted under an inverted microscope (Olympus).

**Wound healing assay**

For the cell migration *in vitro*, 48 h after transfection, cells were seeded in 6-well plates with Culture-Insert 2 Well (µ-Dish 35 mm, high ibiTreat) according to the manufacturer’s instruction and then incubated in medium containing 2% FBS. Images were captured 0, 12, and 24 h in the same field under magnification after the culture-insert removed to assess cell migration using a light microscope (Olympus).

**Colony formation assay**

For the colony formation assay, cells (800 cells/well) were seeded into 6-well culture plates 48 h after transfection and incubated for 2 weeks. The colonies were fixed in 4% formaldehydum polymerisatum and stained with 0.1% crystal violet. Colonies containing ≥50 cells were counted.

**Flow Cytometry and TUNEL assay**

For the cell apoptotic rate, cells were harvested 48 h after transfection, digested with 0.25% trypsin, centrifuged, and washed once with cold PBS. Apoptosis was detected using an Annexin V-FITC/PI kit (BD Biosciences) according to the manufacturer's instructions. Briefly, cells were suspended in 500 µl of 1× binding buffer containing 5 µl of Annexin V-FITC and 5 µl of PI for 30 min in the dark. The cell apoptotic rate of each group was assessed by a flow cytometer (CoulterNavios EX, Beckman).

**Western blotting**

For western blots, samples were separated on SDS–PAGE gels and then transferred to PVDF membranes (Millipore). Membranes were processed according to the ECL western blotting protocol (GE Healthcare). The following antibodies were used in western blots: anti-CUEDC2 (Sangon, D155297), anti-PTEN (Sangon, D261095), anti-P-AKT (Proteintech, 66444-1-Ig), anti-AKT (Proteintech, 10176-2-AP), anti-P21 (Proteintech, 10355-1-AP), β-actin (Sangon, D110001). Antibody validation is provided on the manufacturers’ websites.

**Lentivirus production and generation of stable cell lines**

Full-length BCYRN1 or empty vector (control vector) was synthesized and inserted into the pSin-EF2-puro retroviral vector by Hanbio (Hanbio). Both retroviral vectors were transfected into the U251 cell line and 48 h post-transfection, the cells were selected with puromycin (4 μg/mL) for 2 weeks to construct cell lines with stably expressing BCYRN1 or carrying the vector alone.

***In vivo* tumor xenograft assay**

BALB/c nude mice (male, 4 weeks of age) were purchased from Charles River Labs (Beijing, China) and maintained under specific pathogen-free conditions. A total of 1 × 10^7^ logarithmically growing U251 cells with or without BCYRN1 overexpression were resuspended in 0.2 ml PBS and subcutaneously injected into the right side of mice in each group (5 mice for each group) after acclimatization for 1 week. Tumor volume was measured every 3 days when the tumors were palpable, and the volumes were calculated using the formula: V = 0.5 × length × width^2^. Mice were sacrificed by cervical dislocation after 17 days, then the tumors were removed, weighed and used for immunohistochemistry assays. Bioluminescent imaging was performed using the IVIS Lumina LT Series III Imaging System (IVIS Lumina) with administration of D-luciferin (150 mg/kg i.v.). Tumors were fixed in formalin and embedded in paraffin using a conventional method for further histological hematoxylin and eosin (H&E) staining. All animal studies were conducted with the approval of the ethics committee.

**RNA pulldown with biotinylated antisense oligonucleotides**

RNA pulldown with 5’-biotinylated antisense oligos was modified from a previously described method [10]. In brief, cells were washed with cold PBS once, then cross-linked in a UV cross-linker (UVP) at 1200mJ strength. The cells were scraped and resuspended in RIPA buffer (50 mM Tris-Cl, pH 8.0, 150 mM NaCl, 5 mM EDTA, 1% NP-40, 0.1% SDS, 1 mM DTT, Complete protease inhibitor, and 0.1 U/µl RNase inhibitor) for 10 min on ice, then harvested and sonicated for 10 min. Then, samples were centrifugated at 13,000 rpm for 20 min. 100 pmol probes were added to the supernatant at 4 °C for 2 h. Streptavidin Dynabeads beads (M-280, Invitrogen) were washed three times with RIPA buffer and supplemented with 1 mg/ml BSA and 0.5 mg/ml yeast tRNA rotated for 1 h. The washed/blocked beads were added into the supernatant with probes and then rotated for 4 h at 4 °C. After washing three times with RIPA buffer supplemented with 500 mM NaCl, beads were harvested with magnets (Life Technologies). RNAs and proteins were eluted from beads for further analysis.

**RNA immunoprecipitation (RIP)**

RIP was carried out as previously described [10]. The following antibody was used: anti-AGO2 (Proteintech, 10686-1-AP). Antibody validation is provided on the manufacturers’ websites.

**Dual luciferase reported assay**

Potential binding sites were predicted with TargetScan database (<http://www.targetscan.org>). CUEDC2 fragments containing the predicted wild-type (wt) or mutant (mut) miR-619-5p binding sites were synthesized and cloned into the pmiR-RB-ReportTM (RiboBio). The 293T cells were cultured in 24-well plates and transfected with 200 ng luciferase reporter plasmid containing either wide-type or mutant 3’UTR of CUEDC2 by Lipofectamine3000 reagent (Invitrogen). Relative luciferase activity was measured 48 h post-transfection using the Dual Luciferase Reporter Assay system (Promega) according to the manufacturer’s instruction. Firefly luciferase activity was normalized to the corresponding Rellina luciferase.

**GO Analysis**

The GO enrichments of the significantly dysregulated mRNAs in co-expression network were analyzed by GOrilla web-server with the background of all mRNAs detected in normal and GBM tissues.

**Data deposition**

RNA-sequencing data have been deposited in the Gene Expression Omnibus (GEO) database under accession number GSE153692.

**Statistical Analysis**

All statistical analyses were performed with the SPSS 20.0 statistical software package. Data are expressed as mean ± SD from at least three independent experiments. Differences were evaluated by Student’s t test for two groups, one-way analysis of variance for multiple groups, and parametric generalized linear model with random effects for tumor growth. P values <0.05 were considered statistically significant and all statistical tests asterisks indicate statistical significance.

**Supplementary Figure legends**

**Figure. S1** Three cases of glioma patients and identification of abnormal lncRNAs. **a** Three glioma patients were diagnosed by enhanced MRI. **b** WHO classification and histological types of three glioma patients. **c** Hierarchical cluster analyses of the differential expressed lncRNAs in three normal and glioma tissues. Red color indicates high expression level, and blue color indicates low expression level. **d** Six dysregulated lncRNAs identified from RNA-seq were validated in 10 normal tissues and 21 GBM tissues by RT-qPCR. N, normal tissues. G, glioma tissues. Error bars, S.E.M. from three independent experiments. *p< 0.05; ****p< 0.0001by two-tailed Student’s *t*-test.

**Figure. S2** BCYRN1 inhibits glioma progression *in vitro*. **a** Overexpression level of BCYRN1 in T98G cells. **b** Expression level of BCYRN1 in U251 cells treated with two independent siRNAs. **c, d** CCK-8 analysis of glioma cells with overexpression or silencing of BCYRN1. **e, f** Colony formation assays of glioma cells with overexpression or silencing of BCYRN1. **g, h** Flow cytometry of glioma cells with overexpression or silencing of BCYRN1. **i, j** TUNEL assays of glioma cells with overexpression or silencing of BCYRN1. Scale bars, 50μm. **k, l** Transwell assays of glioma cells with overexpression or silencing of BCYRN1. **m, n** Wound-healing assays of glioma cells with overexpression or silencing of BCYRN1. Error bars, S.E.M. from three independent experiments. *P< 0.05; **P< 0.01; ***P< 0.001 by two-tailed Student’s *t*-test. #P was used to show the significance between NC and Si-BCYRN1-2.

**Figure. S3** Characterization and function of miR-619-5p. **a** Expression level of miR-619-5p were validated in 30 normal tissues and 30 glioma tissues (2 Grade I, 9 Grade II, 5 Grade III, 14 Grade IV) by RT-qPCR. **b** RT-qPCR analysis of the relative expression of miR-619-5p in five glioma cell lines. **c** Efficiencies of biotinylated RNA pulldown in glioma cells. **d** Expression level of miR-619-5p in T98G cells after transfection with miR-619-5p mimic. **e** Expression level of miR-619-5p in U251 cells treated with miR-619-5p inhibitor. **f** , **g** CCK-8 analysis of glioma cells with overexpression or silencing of miR-619-5p. **h**, **i** TUNEL assays of glioma cells with overexpression or silencing of miR-619-5p. Scale bars, 50μm. **j, k** Transwell assays of glioma cells with overexpression or silencing of miR-619-5p. Error bars, S.E.M. from three independent experiments. *P< 0.05; **P< 0.01; ***P< 0.001; ****p< 0.0001 by two-tailed Student’s *t*-test.

**Figure. S4** Biological functions of miR-619-5p in glioma were rescued by BCYRN1. **a** Expression level of miR-619-5p in U251 cells treated with miR-619-5p mimic and co-transfection with BCYRN1 overexpression vector. **b** Expression level of miR-619-5p in T98G cells treated with miR-619-5p inhibitor and co-transfection with BCYRN1 siRNAs. **c** CCK-8 analysis in T98G cells transfected with miR-619-5p inhibitor alone or co-transfection with BCYRN1 siRNAs. **d** Colony formation assays in T98G cells transfected with miR-619-5p inhibitor alone or co-transfection with BCYRN1 siRNAs. **e** Flow cytometry assays in T98G cells transfected with miR-619-5p inhibitor alone or co-transfection with BCYRN1 siRNAs. **f** Transwell assays in T98G cells transfected with miR-619-5p inhibitor alone or co-transfection with BCYRN1 siRNAs. **g** Wound-healing assays in T98G cells transfected with miR-619-5p inhibitor alone or co-transfection with BCYRN1 siRNAs. Error bars, S.E.M. from three independent experiments. *P< 0.05; **P< 0.01; ***P< 0.001; ****p< 0.0001 by two-tailed Student’s *t*-test.

**Figure. S5** Characterization of CUEDC2 and its biological functions in glioma were rescued by miR-619-5p. **a** Downregulated CUEDC2 were validated in 30 normal tissues and 30 glioma tissues(2 Grade I, 9 Grade II, 5 Grade III, 14 Grade IV) by RT-qPCR. **b** Kaplan-Meier plots of overall survivals in glioma patients with high (n=58) and low (n=86) levels of CUEDC2 (p=0.049). **c** CCK-8 analysis in T98G cells transfected with CUEDC2 siRNAs alone or co-transfection with miR-619-5p inhibitor. **d**, **e** Transwell assays in T98G cells transfected with CUEDC2 siRNAs alone or co-transfection with miR-619-5p inhibitor. **f, h** The protein expression level of CUEDC2 in T98G cells treated with CUEDC2 siRNAs alone or co-transfection with miR-619-5p inhibitor. **g, i** The protein expression level of CUEDC2 in U251 cells treated with CUEDC2 overexpression alone or co-transfection with miR-619-5p mimic. **j, l** The protein expression level of PI3K/Akt signaling related proteins in T98G cells treated with CUEDC2 siRNAs alone or co-transfection with miR-619-5p inhibitor. **k, m** The protein expression level of PI3K/Akt signaling related proteins in U251 cells treated with CUEDC2 overexpression alone or co-transfection with miR-619-5p mimic. Error bars, S.E.M. from three independent experiments. *P< 0.05; **P< 0.01; ***P< 0.001; ****p< 0.0001 by two-tailed Student’s *t*-test. In **b**, error bars, S.E.M. P-value was calculated by Mantel-Cox log rank test.
