# Supplementary figures and images for: LncRNA BCYRN1 inhibits glioma tumorigenesis by competitively binding with miR-619-5p to regulate CUEDC2 expression and the PTEN/AKT/p21 pathway
Source: Oncogene. 2020 Sep 25;39(45):6879–92. doi: 10.1038/s41388-020-01466-x (PMC7644463; doi:10.1038/s41388-020-01466-x)

Supplementary figure1

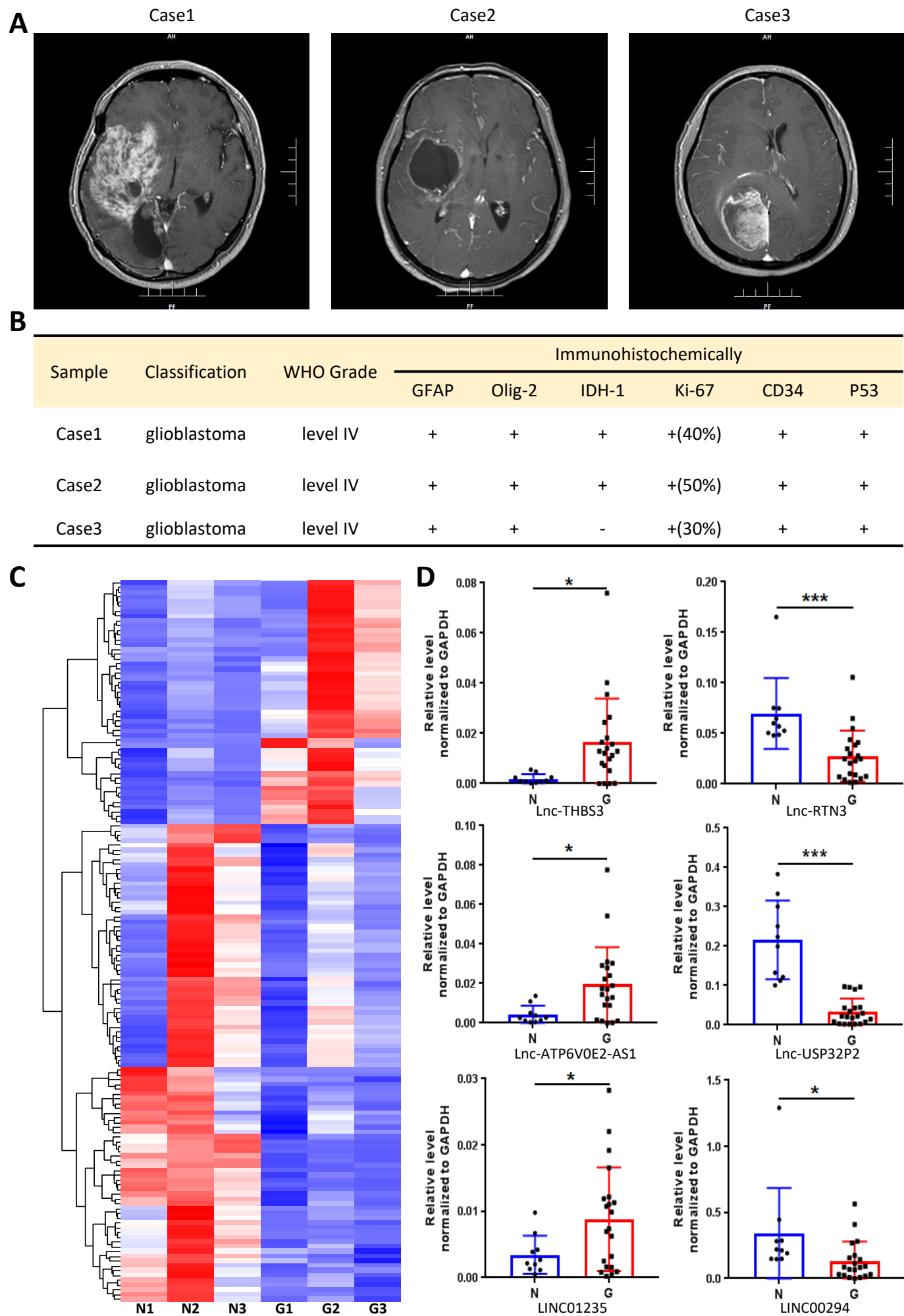

Supplement: Supplementary file 2 — Figure S1 [file 41388_2020_1466_MOESM2_ESM.pdf]

# Supplementary figure2

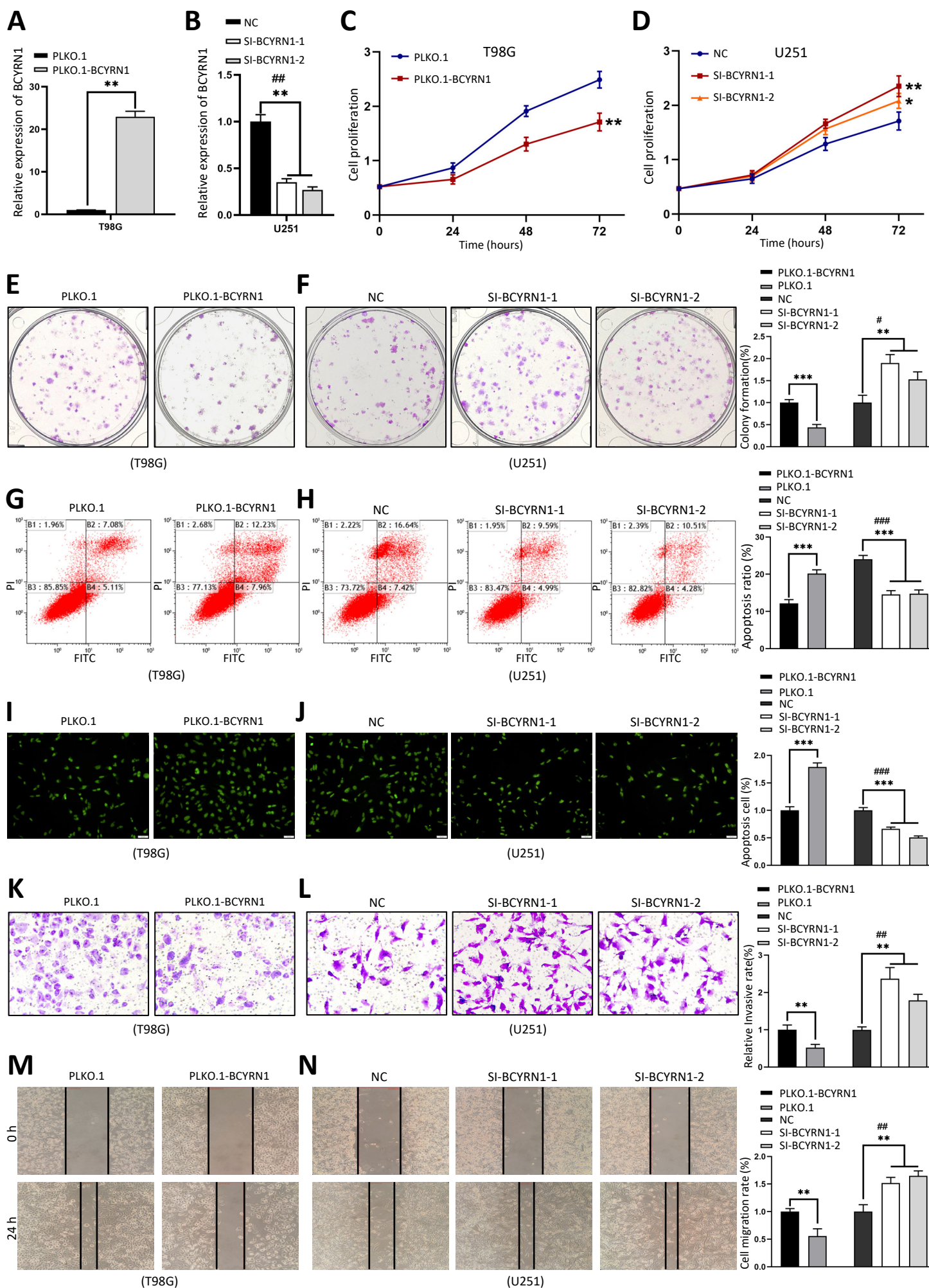

Supplement: Supplementary file 3 — Figure S2 [file 41388_2020_1466_MOESM3_ESM.pdf]

Supplementary figure3

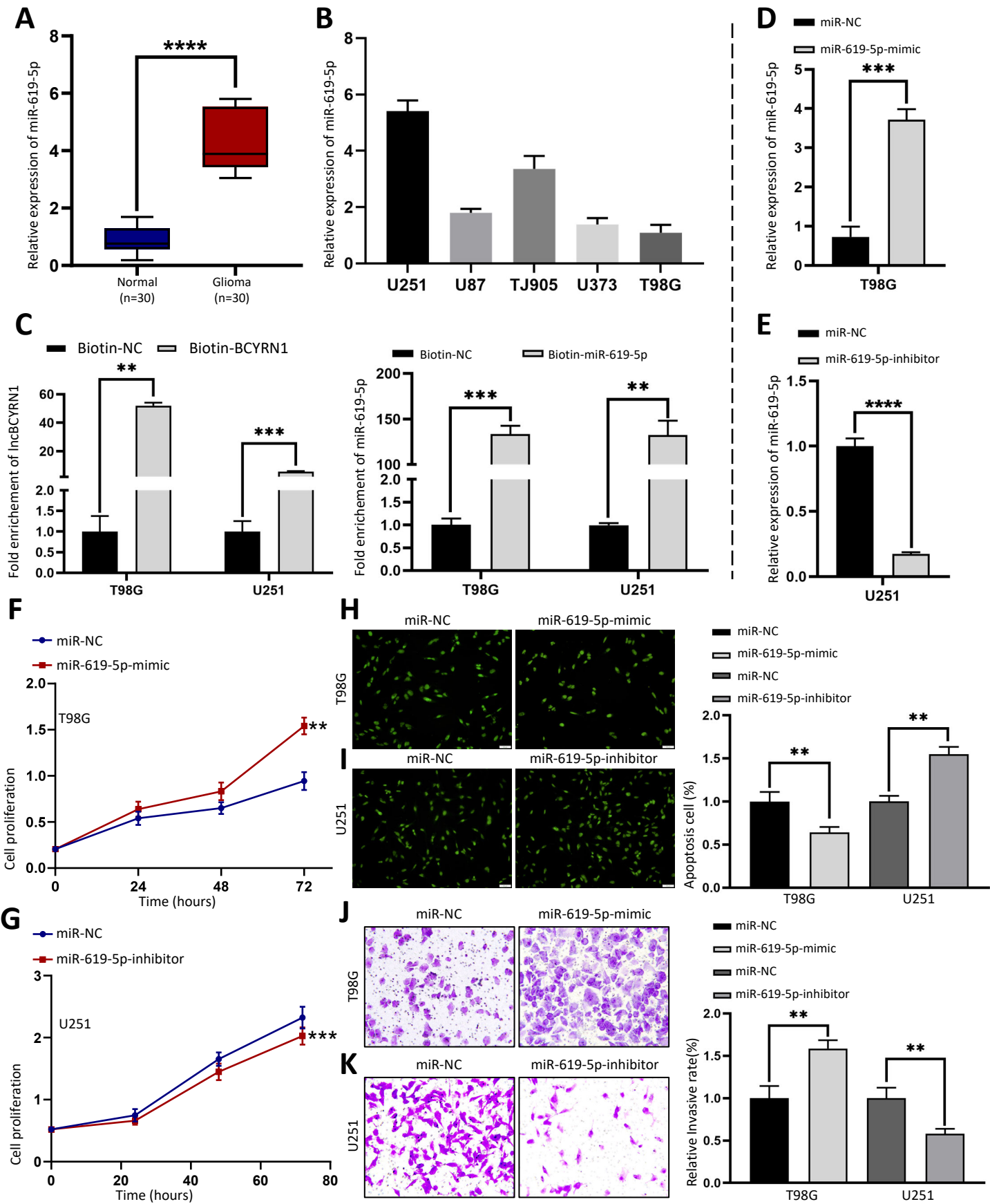

Supplement: Supplementary file 4 — Figure S3 [file 41388_2020_1466_MOESM4_ESM.pdf]

Supplementary figure4

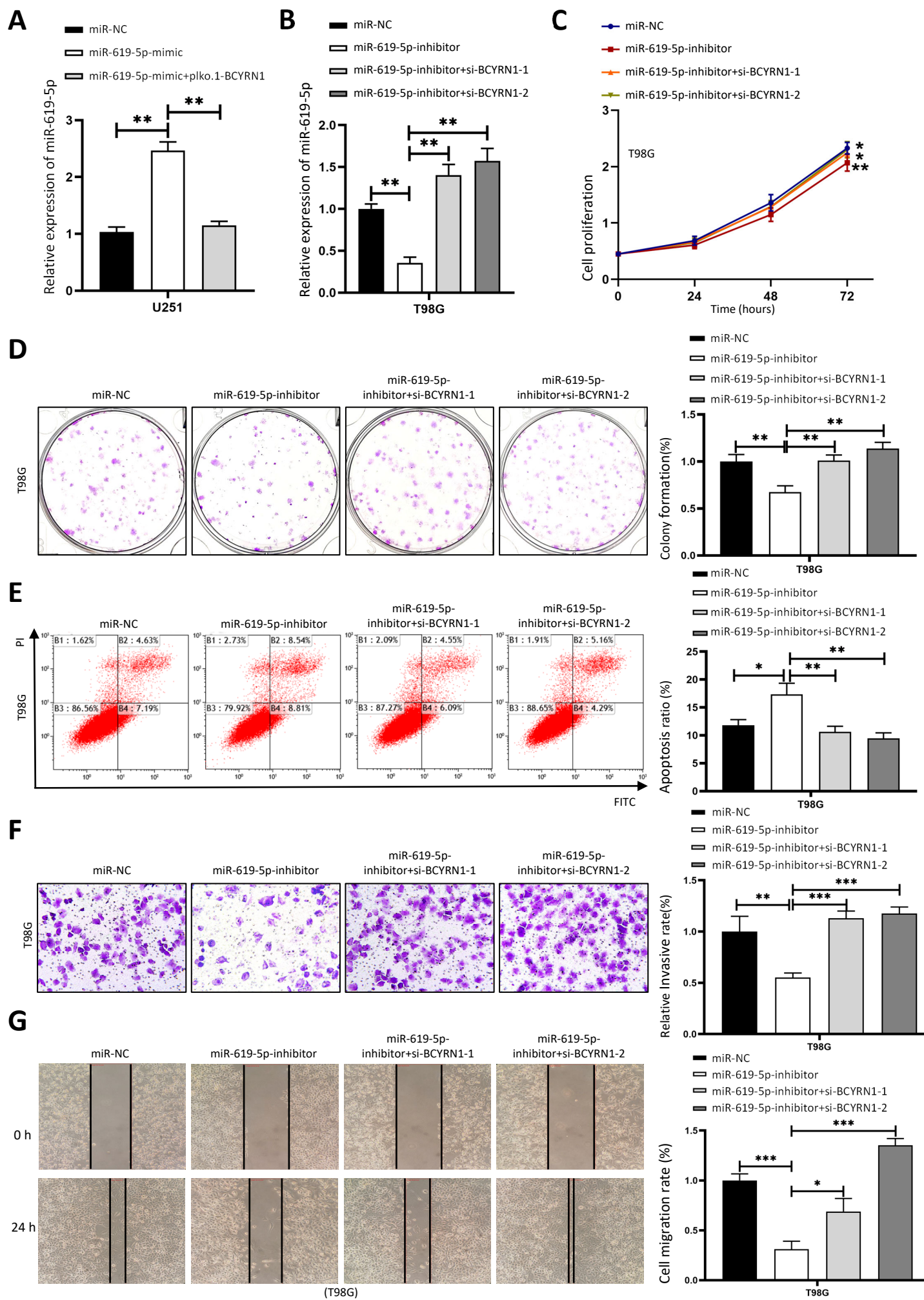

Supplement: Supplementary file 5 — Figure S4 [file 41388_2020_1466_MOESM5_ESM.pdf]

Supplementary figure5

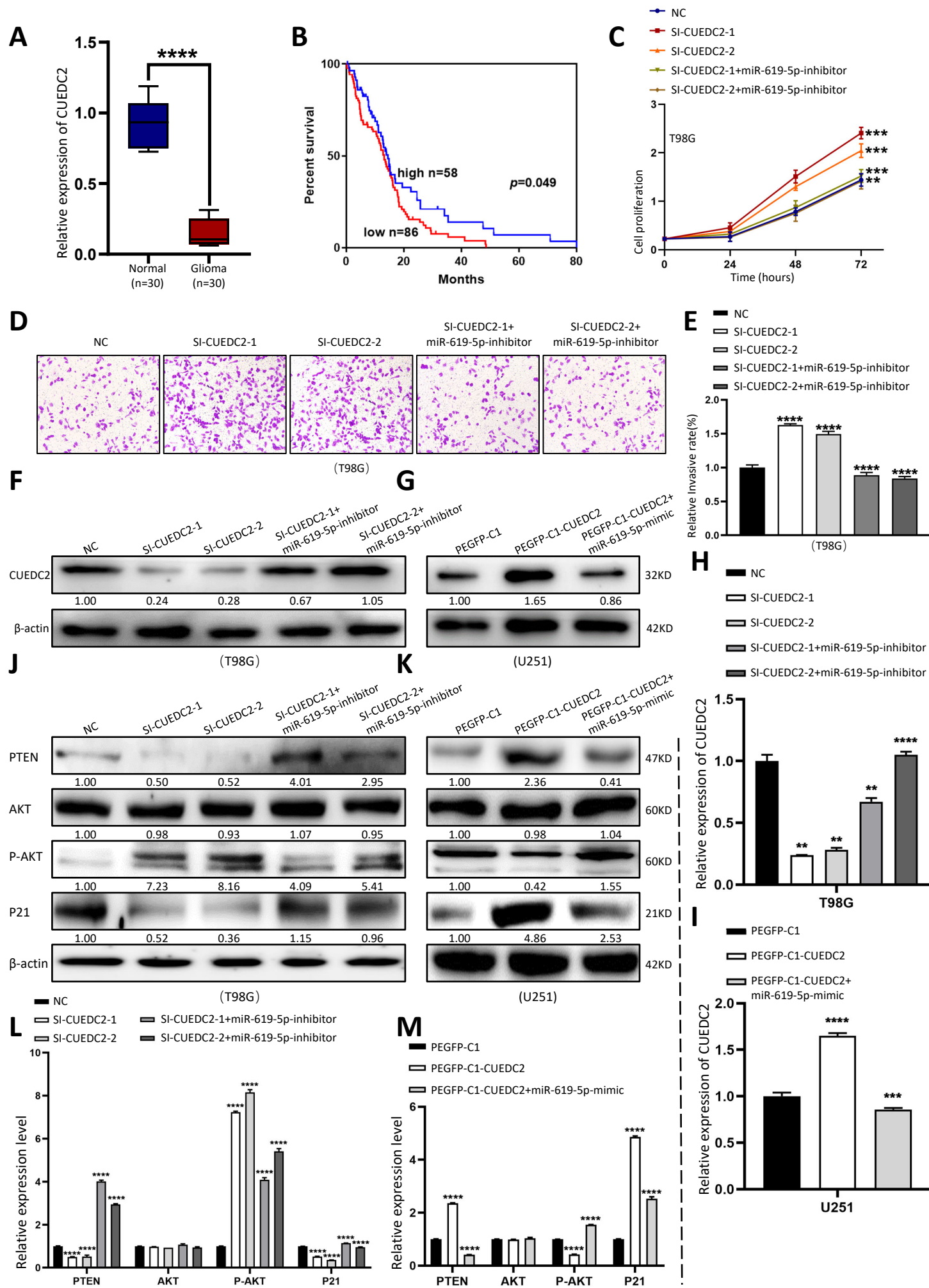

Supplement: Supplementary file 6 — Figure S5 [file 41388_2020_1466_MOESM6_ESM.pdf]
