# Supplementary material for: LncRNA BCYRN1 inhibits glioma tumorigenesis by competitively binding with miR-619-5p to regulate CUEDC2 expression and the PTEN/AKT/p21 pathway
Source: Oncogene. 2020 Sep 25;39(45):6879–92. doi: 10.1038/s41388-020-01466-x (PMC7644463; doi:10.1038/s41388-020-01466-x)
Supplement: Supplementary file 7 — Table S1 [file 41388_2020_1466_MOESM7_ESM.docx]

| **Table S1：Some known transcripts published in the previous studies.** | | | | | | | | | |  |
| --- | --- | --- | --- | --- | --- | --- | --- | --- | --- | --- |
| Genes | N1  RPKM | N2  RPKM | N3  RPKM | | G1  RPKM | G2  RPKM | G3  RPKM | Log2FC | P value | PMID |
| TGF-β2 | 11.78 | 6.42 | 9.36 | 60.17 | | 25.87 | 23.07 | 2.88 | 0.0002 | 29145888 |
| HOXB3 | 0.52 | 0.02 | 0.08 | 16.85 | | 5.32 | 3.46 | 3.90 | 0.0077 | 29456734 |
| FOXM1 | 1.43 | 0.34 | 0.25 | 2.38 | | 7.20 | 3.06 | 2.60 | 0.0096 | 29700308 |
| CD44 | 78.57 | 46.93 | 16.92 | 129.11 | | 84.08 | 139.46 | 2.21 | 0.0070 | 27578526 |
| HCP5 | 8.19 | 4.37 | 2.63 | 22.61 | | 38.91 | 16.10 | 2.12 | 0.0142 | 27434586 |
| PART1 | 28.33 | 2.51 | 28.69 | 2.27 | | 3.11 | 5.66 | -2.69 | 0.0248 | 23726844 |
| MEG3 | 1247.58 | 39.49 | 1720.34 | 50.24 | | 78.91 | 24.17 | -4.64 | 0.0006 | 27306825 |
