# Supplementary material for: LncRNA BCYRN1 inhibits glioma tumorigenesis by competitively binding with miR-619-5p to regulate CUEDC2 expression and the PTEN/AKT/p21 pathway
Source: Oncogene. 2020 Sep 25;39(45):6879–92. doi: 10.1038/s41388-020-01466-x (PMC7644463; doi:10.1038/s41388-020-01466-x)
Supplement: Supplementary file 8 — Table S2 [file 41388_2020_1466_MOESM8_ESM.docx]

| **Table S2 Samples information represented in Figure4b, Figure5b, h, Figure s3a, Figure s5a** | | |
| --- | --- | --- |
| Variable | | Samples (n=30) |
| Sex |  |  |
|  | Male | 17 |
|  | Female | 13 |
| Age(year) |  |  |
|  | ≤45 | 10 |
|  | >45 | 20 |
| WHO grade |  |  |
|  | Ⅰ | 2 |
|  | Ⅱ | 9 |
|  | Ⅲ | 5 |
|  | Ⅳ | 14 |
| Location |  |  |
|  | Frontal | 13 |
|  | Parietal | 2 |
|  | Occipital | 3 |
|  | Temporal | 12 |
| Recurrence |  |  |
|  | NO  Yes | 25  5 |
| Histology |  |  |
|  |  |  |
|  | Oligodendroglioma | 6 |
|  | Astrocytome | 10 |
|  | Glioblastoma | 14 |
