# Supplementary material for: LncRNA BCYRN1 inhibits glioma tumorigenesis by competitively binding with miR-619-5p to regulate CUEDC2 expression and the PTEN/AKT/p21 pathway
Source: Oncogene. 2020 Sep 25;39(45):6879–92. doi: 10.1038/s41388-020-01466-x (PMC7644463; doi:10.1038/s41388-020-01466-x)
Supplement: Supplementary file 9 — Table S3 [file 41388_2020_1466_MOESM9_ESM.docx]

**Table S3 Oligos used in the study**

| LINC01235-F | TGATAGATTCTACAAGGTTG | For LINC01235  RT-PCR | sFig1d |
| --- | --- | --- | --- |
| LINC01235-R | AGTTTGGAAGGGTCATGGAA |  |  |
| LNC-THBS3-F | TGCTTTTTCACATCTGCCAG | For LNC-THBS3  RT-PCR | sFig1d |
| LNC-THBS3-R | AAGGTGGATAAGAGGTAGAT |  |  |
| LNC-RTN3-F | TGCAGCTCCTCCTGTGCGGT | For LNC-RTN3  RT-PCR | sFig1d |
| LNC-RTN3-R | GAGAAGAGCCAGGATGAGGT |  |  |
| LNC-USP32P2-F | AGAGGTTGATGCATAGCTAG | For LNC-USP32P2  RT-PCR | sFig1d |
| LNC-USP32P2-R | AGGTTGATGGGCTCGACTTG |  |  |
| LNC-ATP6V0E2-AS1-F | TGTAACTGACATGGGACTTGTC | For LNC-ATP6V0E2  RT-PCR | sFig1d |
| LNC-ATP6V0E2-AS1-R | AAGTACTAACTGGACTCAAC |  |  |
| LINC00294-F | GATCCAATGACATTTGATCTTG | For LINC00294  RT-PCR | sFig1d |
| LINC00294-R | CGTTTGATCGTAGTACACGTG |  |  |
| LNC-BCYRN1-F | GCCTGTAATCCCAGCTCTCA | For LNC-BCYRN1  RT-PCR | Fig1d,f,g Fig2a,b Fig3b Fig4b,c,d,e,g Fig5g sFig2a,b sFig3c |
| LNC-BCYRN1-R | GCTTTGAGGGAAGTTACGCT |  |  |
| CUEDC2-F | AAGATGTGCGGAACCCTGAG | For CUEDC2  RT-PCR | Fig5b,c,g  sFig5a |
| CUEDC2-R | AGGGCTCCTGCATCCCTCTG |  |  |
| miR-619-5p-F | CACGCAGCTGGGATTACA | For miR-619-5p  RT-PCR | Fig4b,c,d,e,g  Fig5b sFig4a,b  sFig3a,b,c,d,e |
| miR-619-5p-R | CCAGTGCAGGGTCCGAGGTA |  |  |
| miR-619-5p-RT | GTCGTATCCAGTGCAGGGTCCGAGGTATTCGCACTGGATACGACGGCTCA |  |  |
| miR-423-5p-F | CACGCATGAGGGGCAGAG | For miR-423-5p  RT-PCR | Fig4 |
| miR-423-5p-R | CCAGTGCAGGGTCCGAGGTA |  |  |
| miR-423-5p-RT | GTCGTATCCAGTGCAGGGTCCGAGGTATTCGCACTGGATACGACAAAGTC |  |  |
| miR-320d-F | CACGCAAAAAGCTGGGTT | For miR-320d  RT-PCR | Fig4 |
| miR-320d-R | CCAGTGCAGGGTCCGAGGTA |  |  |
| miR-320d-RT | GTCGTATCCAGTGCAGGGTCCGAGGTATTCGCACTGGATACGACTCCTCT |  |  |
| miR-124-3p-F | CACGCATAAGGCACGCGG | For miR-124-3p  RT-PCR | Fig4 |
| miR-124-3p-R | CCAGTGCAGGGTCCGAGGTA |  |  |
| miR-124-3p-RT | GTCGTATCCAGTGCAGGGTCCGAGGTATTCGCACTGGATACGACTTGGCA |  |  |
| miR-103a-3p-F | CACGCAAGCAGCATTGTA | For miR-103a-3p  RT-PCR | Fig4 |
| miR-103a-3p-R | CCAGTGCAGGGTCCGAGGTA |  |  |
| miR-103a-3p-RT | GTCGTATCCAGTGCAGGGTCCGAGGTATTCGCACTGGATACGACTCATAG |  |  |
| miR-107-F | CACGCAAGCAGCATTGTA | For miR-107  RT-PCR | Fig4 |
| miR-107-R | CCAGTGCAGGGTCCGAGGTA |  |  |
| miR-107-RT | GTCGTATCCAGTGCAGGGTCCGAGGTATTCGCACTGGATACGACTGATAG |  |  |
| miR-485-5p-F | CACGCAAGAGGCTGGCCG | For miR-485-5p  RT-PCR | Fig4 |
| miR-485-5p-R | CCAGTGCAGGGTCCGAGGTA |  |  |
| miR-485-5p-RT | GTCGTATCCAGTGCAGGGTCCGAGGTATTCGCACTGGATACGACGAATTC |  |  |
| miR-146b-5p-F | CACGCATGAGAACTGAAT | For miR-146b-5p  RT-PCR | Fig4 |
| miR-146b-5p-R | CCAGTGCAGGGTCCGAGGTA |  |  |
| miR-146b-5p-RT | GTCGTATCCAGTGCAGGGTCCGAGGTATTCGCACTGGATACGACCAGCCT |  |  |
| miR-671-3p-F | CACGCATCCGGTTCTCAG | For miR-671-3p  RT-PCR | Fig4 |
| miR-671-3p-R | CCAGTGCAGGGTCCGAGGTA |  |  |
| miR-671-3p-RT | GTCGTATCCAGTGCAGGGTCCGAGGTATTCGCACTGGATACGACGGTGGA |  |  |
| miR-136-5p-F | CACGCAACTCCATTTGTT | For miR-136-5p  RT-PCR | Fig4 |
| miR-136-5p-R | CCAGTGCAGGGTCCGAGGTA |  |  |
| miR-136-5p-RT | GTCGTATCCAGTGCAGGGTCCGAGGTATTCGCACTGGATACGACTCCATC |  |  |
| miR-15b-5p-F | CACGCATAGCAGCACATC | For miR-15b-5p  RT-PCR | Fig4 |
| miR-15b-5p-R | CCAGTGCAGGGTCCGAGGTA |  |  |
| miR-15b-5p-RT | GTCGTATCCAGTGCAGGGTCCGAGGTATTCGCACTGGATACGACTGTAAA |  |  |
| miR-34a-5p-F | CACGCATGGCAGTGTCTT | For miR-34a-5p  RT-PCR | Fig4 |
| miR-34a-5p-R | CCAGTGCAGGGTCCGAGGTA |  |  |
| miR-34a-5p-RT | GTCGTATCCAGTGCAGGGTCCGAGGTATTCGCACTGGATACGACACAACC |  |  |
| miR-566-F | CACGCAGGGCGCCTGTGA | For miR-566  RT-PCR | Fig4 |
| miR-566-R | CCAGTGCAGGGTCCGAGGTA |  |  |
| miR-566-RT | GTCGTATCCAGTGCAGGGTCCGAGGTATTCGCACTGGATACGACGTTGGG |  |  |
| GAPDH-F | CTTCATTGACCTCAACTACATGG | For GAPDH qPCR | Fig1f |
| GAPDH-R | CTCGCTCCTGGAAGATGGTGAT |  |  |
| U6-F | CGCTTCGGCAGCACATATAC | For U6 qPCR | Fig1f |
| U6-R | TTCACGAATTTGCGTGTCAT |  |  |
| si-NC | UUCUCCGAACGUGUCACGU  ACGUGACACGUUCGGAGAA | Negative control | Fig2d,f,h,j,l,n  Fig4d Fig5i,k  Fig6b,e  sFig2d,e,g,j,k,m  sFig4 |
| si-BCYRN1-1 | UUGCUUUGAGGGAAGUUAC GUAACUUCCCUCAAAGCAATT | siRNAs of BCYRN1 |  |
| si-BCYRN1-2 | UUCCUUUUUCUGGAGAACG CGUUCUCCAGAAAAAGGAATT |  |  |
| si-CUEDC2-1 | CAUCAGAGGAGAACUUCGA  UCGAAGUUCUCCUCUGAUG | siRNAs of CUEDC2 | sFig5c,d,e,h |
| si-CUEDC2-2 | CCAAGAUGAGGCAACUGGCGCUGAG  CUCAGCGCCAGUUGCCUCAUCUUGG |  |  |
| BCYRN1-probe1 | TTTTCTGGAGAACGGGGTCTCGCTATATTG | For lncBCYRN1 RIP with 5’biotin labeled | Fig4g  sFig3c |
| BCYRN1-probe2 | GGGGGTTGTTGCTTTGAGGGAAGTTACGCT |  |  |
| BCYRN1-probe3 | TTAGCCTCCCTGAGAGCTGGGATTACAGGC |  |  |
| Srcamble | TTCTCCGAACGTGTCACGTTCGAACGTGTC | Control probe with 5’biotin labeled |  |
| miR-619-5p-probe | GGCTCATGCCTGTAATCCCAGC | For miR-619-5p RIP with 5’biotin labeled |  |
